# What does a Peer Support Worker do in a Forensic Mental Health Clinic for Addicted Offenders?

**DOI:** 10.1192/j.eurpsy.2023.1868

**Published:** 2023-07-19

**Authors:** P. Walde, B. Völlm

**Affiliations:** Forensic Psychiatry, Rostock University Medical Center, Rostock, Germany

## Abstract

**Introduction:**

Peer support work can be an effective way to support patients and their participation in psychiatric treatment. Unlike in general psychiatry there is less experience with peer support work in forensic mental health inpatient settings. Characteristics different from general psychiatry, e.g., regarding patient structure and background, might lead to different tasks of peer support workers and subjects of conversation.

**Objectives:**

We aim to present an overview of tasks and conversation topics of a peer support worker in a forensic mental health setting for addicted offenders during an 12 month period. We address tasks on regular and irregular basis and the most frequent conversation topics.

**Methods:**

We used the anonymized work documentation about weekly working activities and conversation notes of the peer support worker to extract information. Extracted data were thematically analyzed and clustered into themes for tasks and conversation topics.

**Results:**

Results reveal several recurring and routine tasks, like joining ward rounds and changes of shift or leading a recovery patient group along with one-to-one conversations with patients according to their request. These topics were expanded by irregular tasks like group discussions for special occasions, e.g., after incidents. During one-on-one conversations, patients addressed topics, e.g., about their substance use history, thoughts and issues about their therapy or ways to achieve their goals in the future.

**Conclusions:**

Peer support is a well-accepted offer that can contain various different tasks in groups and in one-on-one settings as well. Although the peer support worker is seated on one ward, there are many requests from other wards, too. There also exists a broad range of conversation topics, some might be also present in general psychiatric wards and others that might be more unique to forensic settings. The broad range of tasks and acceptance of peer support make it necessary to provide corresponding resources like peer support staff and payment.

**Disclosure of Interest:**

None Declared

